# Clinical outcome in patients with acute coronary syndrome and outward remodeling is associated with a predominant inflammatory response

**DOI:** 10.1186/1756-0500-7-669

**Published:** 2014-09-24

**Authors:** Alejandra Madrid-Miller, Luis Chávez-Sánchez, Guillermo Careaga-Reyna, Gabriela Borrayo-Sánchez, Karina Chávez-Rueda, Silvestre Armando Montoya-Guerrero, Arturo Abundes Velazco, Mariano Ledesma-Velasco, María Victoria Legorreta-Haquet, Francisco Blanco-Favela

**Affiliations:** División de Investigación en Salud y Servicio de Hemodinamia de la Unidad Médica de Alta Especialidad, Hospital de Cardiología, Centro Médico Nacional Siglo XXI, Instituto Mexicano del Seguro Social, México, DF México; Unidad de Investigación Médica en Inmunología, Unidad Médica de Alta Especialidad, Hospital de Pediatría, Centro Médico Nacional Siglo XXI, Instituto Mexicano del Seguro Social, Avenida Cuauhtémoc 330, Col. Doctores, CP: 06720 México City, México; Unidad Médica de Alta Especialidad, Hospital General del Centro Médico Nacional la Raza, Instituto Mexicano del Seguro Social, México, DF México; Coordinación de Investigación en Salud, Centro Médico Nacional Siglo XXI, Instituto Mexicano del Seguro Social, México, DF México; Servicio de Hemodinamia, Instituto Nacional de Cardiología, México, DF México

**Keywords:** Acute coronary syndrome, Low-density lipoprotein, Inflammation

## Abstract

**Background:**

Pro-inflammatory molecules and low-density lipoproteins play essential roles in the atherosclerosis. The aim of our study was to establish an association among the cytokines secreted by peripheral blood mononuclear cells and the serum concentration in patients with unstable angina and coronary outward remodeling before and after percutaneous coronary intervention. The clinical and coronary responses were evaluated 6 months after the procedure.

**Findings:**

Twenty-two patients with unstable angina were evaluated prior to after percutaneous coronary intervention and 6 months after procedure by coronary intravascular ultrasound. Eleven of the patients had recurrent angina, while 9 presented restenosis and an increase in the percentage of total plaque area. These 11 patients displayed higher levels of C-reactive protein than those without coronary events (1.27 vs. 0.43 mg/dl, respectively; p = 0.029) and a tendency to increase levels of interleukin (IL)-8 and transforming growth factor-β1, but lower levels of IL-10 (52.09 vs. 141.5 pg/ml, respectively; p = 0.035). Activated peripheral blood mononuclear cells from patients with restenosis presented higher levels of proliferation, CD86 expression and higher IL-1, and increased IL-10 compared to those in patients without restenosis.

**Conclusions:**

Patients with unstable angina and coronary outward remodeling who displayed a pro-inflammatory response experienced recurrent coronary events and an increased percentage of total plaque area. In contrast, better outcomes were observed in patients with anti-inflammatory responses. This response could be secondary to low-density lipoproteins.

## Findings

### Background

The development and progression of coronary atherosclerotic lesions are characterized by lipid accumulation, cellular proliferation, and an inflammatory response, all of which causes changes in the vascular arterial wall [1]. These processes determine the plaque vulnerability, severity of lumen obstruction, and homodynamic and ischemic myocardial repercussion [2, 3]. Necropsy studies have demonstrated that lesion with positive or outward remodeling has higher lipid contents, as well as macrophage and other inflammatory cells counts, both markers of plaque vulnerability [4]. It seen that in patients with acute coronary syndrome, lipid accumulation in a culprit lesion is related with vascular expansion in atherogenesis. Additionally, remodeling of the arterial wall is an important mechanism in restenosis after percutaneous coronary interventions [3]. Angiographic follow-up studies have associated inflammatory activity with the frequency of restenosis, supported by the presence of inflammatory cells such as monocytes and T cells in the restenotic lesions [5, 6].

However, until now, the nature of this inflammatory response has been controversial. Some of the possible triggers of the inflammatory response include *Helicobacter pylori*, *Chlamydia pneumoniae* and modified low-density lipoprotein (LDL) cholesterol [1, 7]. Several studies have demonstrated that different forms of oxidized low-density lipoproteins (oxLDL) contribute to the development of atherosclerotic lesions through an inflammatory response [8, 9], supporting the idea that oxLDL may be a key antigen in atherosclerosis [7].

In addition, some studies have demonstrated that oxLDL activates different cell types and induces the secretion of pro-inflammatory interleukins (IL) such as IL-1β and IL-6 [10]. Others have shown that the T cells from patients with unstable angina (UA) can be activated with oxLDL; in contrast, T cells from stable patients exhibit a lower response to oxLDL [11]. Furthermore, native LDL (nLDL) increases the generation of vascular endothelial superoxide anions in situ, suggesting that it plays a role in the premature development of atherosclerosis [12].

However, specific mechanisms and temporal course of the complex interplay between mechanical dilatation, inflammatory response and corresponding changes in arterial anatomy and physiology are still poorly understood.

The aim of this study was to determine whether there is an association between the type of cytokines secreted from peripheral blood mononuclear cells (PBMCs) activated with nLDL *in vitro* and the serum cytokines concentrations, from patients with unstable angina and coronary outward remodeling before and after intravascular ultrasound-guided percutaneous coronary intervention (PCI), and the clinical and coronary responses were evaluated 6 months after the procedure.

## Methods

### Patient population

This study included 22 patients with unstable angina who were admitted to the Coronary Care Unit of the Hospital de Cardiología, Centro Médico Nacional “Siglo XXI”. All of the patients were stable and had not experienced angina within the 48 hours before the procedure. Informed consent was obtained from all patients and healthy donors. The study was approved by the Human Ethics and Medical Research Committee of the Instituto Mexicano del Seguro Social (IMSS) and was conducted according to the Helsinki Declaration guidelines. The inclusion criteria included the following: a) patients younger than 75 years of age; b) angina duration less than 30 minutes, associated with an ST-segment depression of more than 1 mm or dynamic T wave changes at rest electrocardiogram during angina and no evidence of myocardial infarction detected with enzyme markers (MB creatine kinase and troponin I); c) patients eligible for percutaneous coronary intervention (PCI) with a bare metal stent implantation; d) it was necessary to confirm the presence of positive (or outward) artery remodeling at the culprit lesion with intravascular ultrasound images before coronary intervention. The exclusion criteria included the following: patients with recent bypass surgery or previous PCI; patients with left bundle branch block that invalidated ST-segment analysis, known malignancies, hematological and immunological disorders, any other inflammatory conditions or infections likely to be associated with the acute phase response, previous immunosuppressive or anti-inflammatory therapy, serum creatinine ≥1.5 mg/dl or known allergic reactions to iodine contrast medium. All patients received optimal anti-ischemic therapy (dual antiplatelet therapy with aspirin and clopidogrel, unfractionated heparin, intravenous nitroglycerine, beta blockers and statins), calcium channel blockers and ACE inhibitors, as required for each patient. All patients underwent laboratory tests, including those to measure total cholesterol, triglycerides, high density lipoprotein cholesterol, cholesterol LDL, white blood cell counts, creatinine and C-reactive protein.

Twelve healthy, normolipemic, 30- to 40-year-old volunteers without cardiovascular risk factors or clinically apparent atherosclerotic disease were also included as controls.

### Coronary angiography and intravascular ultrasound

All patients underwent coronary angiography under the same conditions. The culprit lesion was defined by a significant stenosis >50%, abnormal thrombolysis in a myocardial infarction (TIMI) flow grade of 1 or 2, or an intra-luminal defect strongly suggestive of a thrombus in the vessel lesion. The restenosis was defined as stenosis of ≥50% observed at the 6-month follow-up of the target lesion.

Intravascular ultrasound (IVUS) studies were performed using a commercially available CVI-INSIGHT system (Cardiovascular Imaging Systems, Inc.). In brief, this system consisted of a single-element 40 mHz transducer mounted on the tip of a flexible beveled shaft within a 3.2 Fr short monorail polyethylene imaging sheath to allow the formation of planar images in real time. Prior to the IVUS studies, all patients received intracoronary nitroglycerine (200 μg). The IVUS catheter was advanced 10 mm distal to the culprit lesion site. Studies were recorded for off-line analysis, as previously reported [3, 13]. This recording was used to identify the corresponding image on the postintervention and control studies at the 6-month follow-up. The IVUS studies were performed in the same angiographic projection of the culprit lesion, and with data collected from the axial landmark (aorto-ostial junction to the target lesion), as reported previously [13].

### Quantitative coronary analysis

Quantitative IVUS analysis included measurements of the external elastic membrane (EEM), cross-sectional area (CSA), lumen CSA, CSA of the plaque plus media (P&M), percent plaque area and cross-sectional narrow (CSN). Measurements were taken at the site of the target lesion and in a proximal segment of the culprit artery, and the analysis was performed as previously reported [13]. The EEM CSA at the culprit lesion was compared to a proximal reference with the most normal-looking CSA (within 10 mm proximal). Outward remodeling was defined as lesion external elastic membrane (EEM) cross-sectional area (CSA) greater than the proximal reference.

### Percutaneous coronary intervention

PCI with bare metal stent implantation in the target lesion was guided with IVUS. Procedure was considered successful if the final percentage of the residual stenosis was <30% with a TIMI flow grade of 3 in the absence of recurrent myocardial ischemia, myocardial infarction, need of urgent coronary bypass surgery or death during the hospitalization period. A 6-month follow-up was performed in all patients unless the patient presented clinical indications for coronary angiography before this period of time. All of the angiograms and IVUS images were re-analyzed by two experienced, blinded cardiologists. An inter-observer variability with a kappa proportion of 0.83 was observed.

### LDL isolation

Native LDL (nLDL) (density 1.019-1.063 g/ml) was isolated by sequential ultracentrifugation from the plasma of UA patients who were not candidates for PCI and from normolipemic donors. The plasma density was adjusted to 1.2 g/ml by the addition of solid KBr (J.T. Baker, Phillipsburg, NJ, USA). The plasma solution was then distributed into polycarbonate centrifuge tubes, and a discontinuous density gradient was created by overlaying the plasma solution (3 ml) with 3 ml each of the following densities: 1.065 g/ml, 1.020 g/ml, and 1.006 g/ml. The tubes were ultracentrifuged in a Beckman LX-90 ultracentrifuge equipped with an SW 40 Ti rotor at 30,000 rpm for 22 hours at 4°C. After ultracentrifugation, the low-density lipoprotein was removed. The LDL fraction was dialyzed against phosphate-buffered saline (PBS) containing 0.01 M phosphate (J.T. Baker, NJ, USA), 0.5 M NaCl (Caledon Laboratories Ltd, Ont, Canada), and 0.5 mM EDTA (Sigma, St. Louis, MO, USA), pH 7.4, for 24 hours at 4°C. The degree of oxidation of nLDL from patients and healthy donors was assessed by measuring thiobarbituric acid-reactive substances as previously described [14].

### Flow cytometry

PBMCs were isolated from UA patients (before and 6 months after PCI) by density centrifugation using Lymphoprep (Axis-Shield, Liverpool, UK). The PBMCs were recovered from the interface, washed three times with PBS (pH 7.4) and resuspended in RPMI (Invitrogen, Carlsbad, CA, USA) supplemented with 10% fetal bovine serum (FBS) (Invitrogen, Carlsbad, CA, USA). Subsequently, the PBMCs (2×10^5^/ml) were treated with 10 μg/ml of nLDL obtained from patients and healthy donors. As a negative control, PBMCs were cultured with RPMI (Invitrogen) supplemented with 10% FBS (Invitrogen). For a positive activation control, PBMCs were plated at 2×10^5^/ml in 96-well flat-bottomed tissue culture plates coated with anti-CD3 (BD Biosciences, CA). Regardless of treatment, all cells were incubated for 18, 24, 72 and 168 hours at 37°C. The PBMCs were then stained with antibodies specific for CD4, CD25, CD69, and CD86 or with isotype-control antibodies (all from BD Biosciences) for 20 minutes in the dark at 4°C. The cells were then washed three times with PBS, fixed with 2% paraformaldehyde, and analyzed using a FACS Calibur flow cytometer (BD Biosciences).

### Proliferation assays

PBMCs of UA patients were isolated prior to PCI and 6 months after PCI by density centrifugation using Lymphoprep (Axis-Shield). The PBMCs were recovered from the interface, washed three times with PBS (pH 7.4) and resuspended in RPMI (Invitrogen) supplemented with 10% FBS (Invitrogen). The PBMCs (2×10^5^/ml) were then treated with 10 μg/ml of nLDL obtained from patients and healthy donors. As a negative control, PBMCs were cultured with RPMI (Invitrogen) containing 10% FBS (Invitrogen) and for a positive control, the cells were cultured with anti-CD3 (BD Biosciences). After 3 days, the cells were pulsed with 1 uCi [^3^H] thymidine (GE Healthcare, Uppsala, Sweden) for an additional 3 days. The PBMCs were harvested into glass fiber filters, and the [^3^H] thymidine (GE Healthcare) incorporation was measured using a scintillation counter (Packard 1900TR counter).

### Cytokine assay

PBMCs (1×10^6^/ml) from patients with UA (isolated prior to PCI and 6 months after procedure) were incubated with 10 μg/ml nLDL obtained from patients and healthy donors. Concanavaline A (Sigma) was used as a positive control. As a negative control, the PBMCs were cultured with medium alone. The culture supernatants were collected after 18, 24 and 72 hours and 7 days of incubation at 37°C. Additionally, sera were obtained from patients (prior to PCI and 6 months after PCI) for the measurement of cytokines. The levels of IL-1, IL-8, IL-10, and TGF-β in the supernatants and sera were analyzed using commercial enzyme-linked immunosorbent assay (ELISA) kits (Biosource International, Camarillo, CA, USA) according to the manufacturer’s instructions.

### Statistical analysis

Because the results were not normally distributed, were expressed as medians and percentiles or ranges. Depending on the results obtained from the PCI, the patients were divided into two groups, those with recurrent angina or restenosis and those without recurrent events. Qualitative variables (dichotomy variables) were analyzed using the Chi-square test or Fisher’s exact test. Nonparametric Wilcoxon tests were performed for quantitative paired variables (before and 6 months after PCI). Mann–Whitney U tests were used to compare independent groups (patients with and without recurrent angina or restenosis), and the association between inflammatory markers and clinical outcome was tested using Spearman’s rho correlation coefficient. A one-tailed p-value of ≤0.05 was considered significant with a 95% CI.

## Results

### Patient characteristics

The patients’ median age was 61 years (range: 50 to 73 years old), and the sample included 14 men and 8 women. Of these, 7 had diabetes, 10 had systemic arterial hypertension, 14 were smokers, and 11 had dyslipidemia. All patients had experienced angina events previous to hospitalization, and 8 had prior myocardial infarctions. All patients were admitted with unstable angina considered intermediate or high risk.

### Intravascular ultrasound imaging

#### Correlation of angiographic results with clinical presentation and IVUS results

The coronary angiography showed that 12 patients had single-vessel coronary disease, 8 had 2-vessel disease, and 2 had 3-vessel disease. The anterior descending artery was the most frequent culprit vessel (17 patients), while the culprit was the left circumflex in 3 cases and the right coronary in 2 patients. The most frequent target lesion type was B (5 patients with type B1 and 12 with type B2); in 5 cases we identified a type C lesion. No significant differences in the type and severity of the coronary artery diseases between patients with or without recurrent ischemic events were observed. Qualitative analysis with IVUS showed that fibrolipidic-like atherosclerotic lesions were observed in the culprit vessel (11 cases) more often than lipidic (6 cases) or fibrotic (5 cases) lesions. In 9 patients, the imaging suggested calcium plaque (less than 90° of the vessel circumference), with severe calcium deposition (more than 180°) in just 1 patient. Quantitative analysis was performed for all patients. The median EEM CSA was 16.3 mm^2^ (range: 10.5-25.7 mm^2^). The median vessel diameter was 4.5 mm (range: 3.5-5.7 mm). The median lumen CSA was 2.2 mm (range: 1.8-2.8 mm), and the median CSN was 72.2% (range: 54.3- 83.8%). There were no differences observed between patients with recurrent angina or restenosis and patients without recurrent ischemia at the time of follow-up. Only 1 patient presented coronary spasm as a complication of IVUS, and this was resolved with an intracoronary administration of verapamil and nitroglycerine. Angiographic success was obtained in all patients after the procedure, only 2 patients required 2 stents due to the length of the atherosclerotic plaque, but was not associated with recurrent ischemic events.

At the 6-month follow-up, 11 patients had recurrent angina. Of these, we identified restenosis of the target lesion by coronary angiography in 9 cases (left descending artery in 8 patients and circumflex in 1 patient). The cardiovascular risk factors and TIMI risk score were similarly distributed among patients with a new angina episode and patients without events. Systemic hypertension was the only risk factor that was significantly associated with recurrent ischemic events (p = 0.027, RR = 7.0, 95% CI 1.02 - 47.81) (Table 1). No significant differences were found by quantitative IVUS analysis of the EEM CSA at 6 months. We found a significant increase (p = 0.015) in the plaque percentage area in patients with restenosis (60.83% [50.9-70.6%]) compared to those without restenosis (48.25% [42.8-60%]). The patients with restenosis also had significantly higher (p = 0.075) CSN gains (8.48% [19.60 a – 11.8%]) than those without restenosis (4.25% [23.9 – 3.2%]) and in two patients was identified late stent malposition (Table 2). In addition, the patients with restenosis presented important neointimal hyperplasia (data not shown).

**Table 1:** **Distribution of demographic characteristics and risk factors between patients**

	Recurrent agina or restenosis	
	Yes (n=11)	No (n=11)
Age (years)	63 (50-70)	57 (55-73)
Male	7	7
Hypertension	7*	1*
Diabetes	5	2
Smoking	8	5
Hyperlipidemia	6	5
Previous angina	7	7
Previous MI	3	2
Ejection fraction %	58.2 (49-75)	51 (40-75)
Angiography		
1 vessel	7	5
2 vessels	3	5
3 vessels	1	1

Systemic hypertension was the only risk factor that was significantly associated with recurrent ischemic events (*p = 0.027).

**Table 2:** **Quantitative IVUS image analysis in patients**

	Recurrent angina or restenosis	
	Yes	No
**Before PTCA**		
**EEM CSA (mm** ^**2**^ **)**	16.4 (10.5-25.7)	16. (12.5-18.9)
**Lumen CSA (mm** ^**2**^ **)**	4.23 (3.10-6.40)	4.95 (3.4-6.4)
**Luminal diameter (mm)**	2.1 (1.8-2.8)	2.3 (2.0-2.6)
**CSN (%)**	76.65 (57.4-83.8)	64.6 (54.3-77)
**Lesion length (mm)**	19.5 (15.1-22.2)	19.1 (12.7-21.9)
**After PTCA**		
**EEM CSA (mm** ^**2**^ **)**	17.1 (10.2-25.7)	16.6 (12.8-21.4)
**Lumen CSA (mm** ^**2**^ **)**	8.9 (5.8-16.5)	8.95 (6.3-11.5)
**Luminal diameter (mm)**	3.15 (2.8-4.5)	3.25 (2.8-3.7)
**CSN (%)**	45.4 (36.10-62.3)	48.13 (43.7-62.7)
**Lumen area gain (mm** ^**2**^ **)**	5.05 (1-10)	3.75 (2.8)
**Lumen diameter gain (mm)**	1.10 (0-2)	0.91 (0-2)
**6 months**		
**Outward remodeling**	5 pac.	4 pac.
**ATV (mm** ^**2**^ **)**	16.80 (10.5-24)	14.9 (10.0-23.5)
**LUMEN CSA (mm** ^**2**^ **)**	5.95 (3.9-9.1)	8.6 (6.0-12.0)
**Luminal diameter (mm)**	2.55 (2.1-3.2)	3.0 (2.5-3.9)
**CSN (%)**	60.83 (50.9-70.6)**	48.25 (42.8-60)**
**Lumen CSA lost (mm** ^**2**^ **)**	-1.40 (-0.2 a -3.2)	-0.55 (-0.2 a -6.6)
**CSN gain**	8.48 (-11.8 a 19.60)	4.25 (-3.2 a 23.9)

The results from quantitative IVUS image analysis are similar between patients with and without recurrent ischemic events during follow-up. A significant increase in percent plaque area (CSN) was observed in patients with restenosis at the 6-month follow-up (**p = 0.015 by Mann–Whitney U test).

### Determination of IL-8, CRP, IL-10, and TGF-β levels in the sera

**Figure 1:**
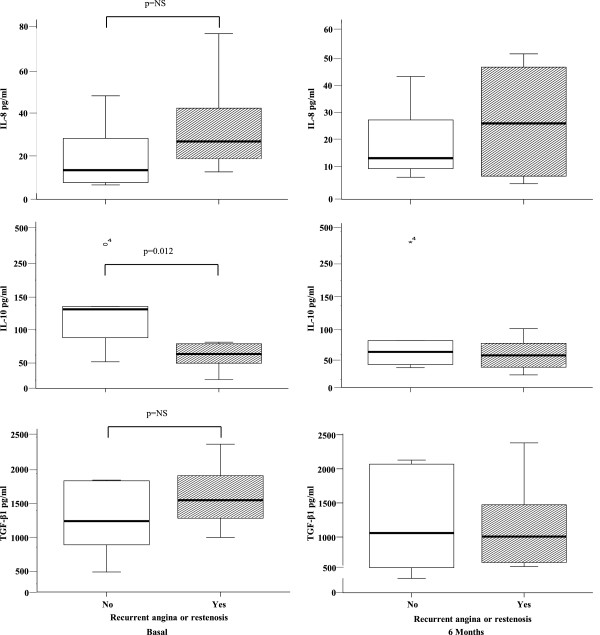
**Comparison of basal and 6-month follow-up serum concentrations of IL-8, IL-10 and TGF β1, in patients with and without recurrent ischemic events or restenosis.** Patients without recurrent events displayed higher levels of IL-10 (p = 0.012) than those with recurrent events. Patients with recurrent events had a tendency to present higher levels of IL-8 than those without recurrent events, but this difference was not statistically significant. No: recurrent angina or restenosis (rectangle open); Yes: with recurrent angina (rectangle closed).

We found a tendency of higher IL-8 concentrations before intervention in patients with recurrent ischemic events when compared to the group of patients without recurrent events, but this difference was not statistically significant (24.19 pg/ml [9.15 - 77.49 pg/ml] in patients with recurrent events vs. 10.01 pg/ml [2.70 - 46.76 pg/ml] in those without recurrent events; correlation coefficient 0.408, p = 0.07). The same tendency was found at the 6-month follow-up (11.9 pg/ml [4.85 - 42.28 pg/ml] in patients with favorable outcomes vs. 24.84 pg/ml [2.40-50.63 pg/ml] in patients with recurrent angina or restenosis, p = NS). Moreover, of the patients presenting elevated levels of C reactive protein, 9 of these had recurrent angina events or restenosis (1.27 mg/dl [0.27-2.3 mg/dl] in patients with recurrent events vs. 0.43 mg/dl, [0.01 - 0.8 mg/dl] in patients without events at 6 months, p =0.029). The IL-10 levels were higher in patients without recurrent events compared to those with recurrent events (141.5 pg/ml [36.47 - 271.30 pg/ml] and 52.09 pg/ml [0.93 - 75.79 pg/ml], respectively; RR 0.25, 95% IC [0.075 – 0.83], p = 0.035). However, at the 6-month follow-up, both groups displayed similar values (55.59 pg/ml [23.54-278.30 pg/ml] in the patients without recurrent ischemic events vs. 48.59 pg/ml [9.03-102.72 pg/ml] in those with recurrent ischemic events, p = NS). Moreover, we found an inverse correlation between the plasma levels of IL-10 and the concentration of CRP (Spearman’s rho correlation coefficient 0.477, p =0.05). In contrast, patients with recurrent ischemic events or restenosis of the target lesion had higher levels of TGF β1 (1139.66 pg/ml [632.43 - 1902.45 pg/ml]) than patients with better outcomes (854.71 pg/ml [161.44 - 1413.83 pg/ml]), although this difference was not statistically significant. At the 6-month follow-up, the TGF β1 levels were similar in both groups of patients (1058.388 pg/ml [392.53- 2128.65 pg/ml] in those with recurrent ischemic events and 1006.49 pg/ml [566.83 - 2380.30 pg/ml] in those without recurrent events, p = NS) (Figure 1).

### Surface expression of CD69, CD25, and CD86 on PBMCs

Before PCI, the CD69 expression was 2.57% [0.98 - 11.51%] on the PBMCs from patients with recurrent angina or restenosis, compared to 1.27% [0.91 - 13.24%] on those from patients without recurrent angina or restenosis (p = NS). The CD25 expression in these groups was 8.5% [0.10 - 16.72%] and 15.06% [6.62 - 25.07%], respectively (p = NS). Similar results were found 6 months after coronary intervention in both groups. In contrast, CD86 expression remained elevated at the 6-month follow-up, principally in those patients with recurrent ischemic events (12.17% [8.03 - 78.92%] vs. 5.15% [3.43-62.56%] in those without recurrent events prior to PCI and 38.38% [7.65-68.14%] in those with recurrent events vs. 10.47% [6.85-44.46%] in those without recurrent events at 6 months following PCI.

### PBMC proliferation in response to LDL

**Figure 2:**
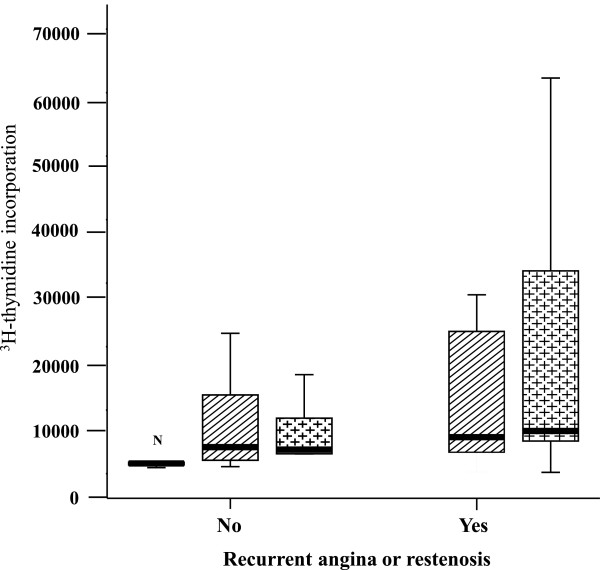
**Proliferative responses to LDL, expressed as [3H] thymidine incorporation.** Comparison of cellular responses from patients without recurrent angina (left) and those with ischemic events (right –YES-). At follow-up, the cells from patients with recurrent angina had a tendency of higher proliferative responses (p = NS). 10 μg/ml of LDLH obtained from healthy donors (rectangle with diagonal lines); 10 μg/ml of LDLP obtained from patients (cross line), N (negative control).

We also investigated the effect of nLDL obtained from patients and healthy donors on the proliferation of PBMCs. As shown in Figure 2, the level of [3H] TdR uptake was relatively low (1800 cmp [145–8,148]) in the negative control. In contrast, in the presence of LDL, the PBMCs of patients without recurrent angina or restenosis showed an increased proliferative response compared with the PBMCs cultured with medium alone. Moreover, the proliferative response to LDL was similar in all patients, although there was a tendency toward higher proliferation levels in the PMBCs from patients with recurrent angina or restenosis compared to those from patients without recurrent angina or restenosis (in response to nLDL obtained from patients: 6,717 cmp [199–62,311] vs. 4,342 cmp [1,215-15,225], respectively; in response to nLDL obtained from healthy donors: 5,992 cmp [260–28,163] vs. 4,653 cmp [1,097-27,245], respectively) (Figure 2).

### Activation of PBMCs in response to LDL

We found that the expression of CD86 on PBMCs was elevated at different times in response to LDL in all patients, particularly in the PBMCs from patients with recurrent angina or restenosis at follow-up (24.94% [0.89-91.63%]), compared to the patients without recurrent events (18.40% [5.71-55.10%]). In contrast, there were no differences in the expression of CD69 or CD25 on the PBMCs of the patients following different lengths of LDL stimulation. There was actually a slight decrease in the expression of CD69 and CD25 following 48 hours of LDL stimulation, and the expression levels were similar at 7 days.

### IL-1 and IL-10 production by LDL-stimulated PBMCs

**Figure 3:**
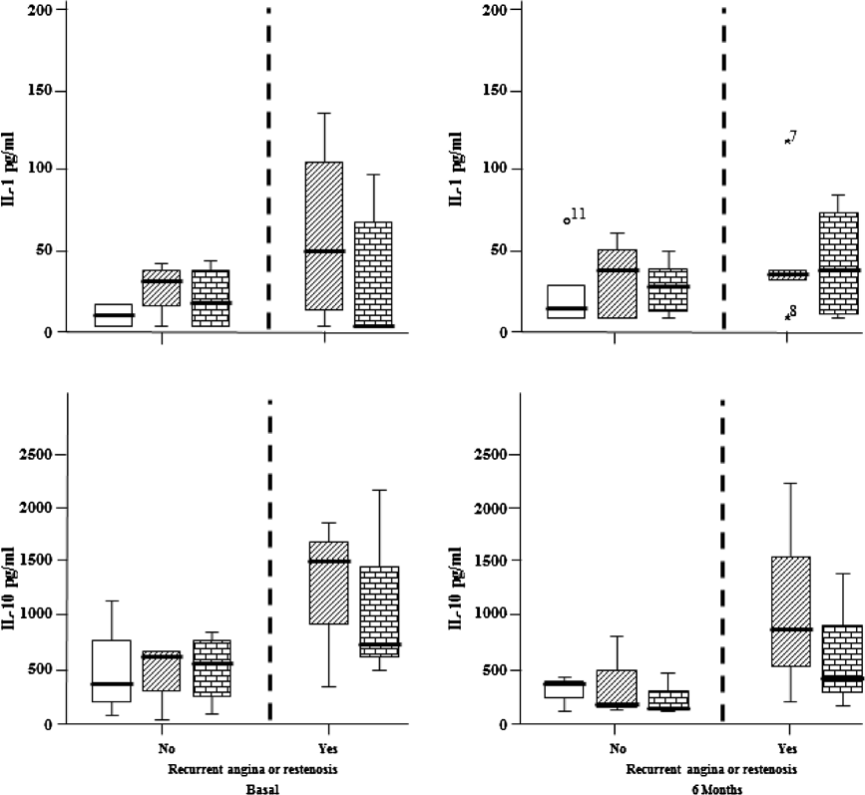
**The interleukin concentrations were compared in the supernatants of cells from patients with recurrent angina or restenosis (left) and from those without recurrent ischemic events (right).** Basal and 6-month follow-up results were also compared. Higher IL-10 concentrations were found in the samples from patients with recurrent events compared to those from patients without recurrent events (Median, 25th and 75th quartile). 10 μg LDLH = 10 μg/ml of LDL obtained from healthy donors (rectangle with diagonal lines); 10 μg LDLP = 10 μg/ml of LDL from patients (rectangle with blocks); W/E: without stimulus (rectangle open).

We found that the *in vitro* nLDL stimulation of PBMCs from patients with or without restenosis resulted in increased IL-1 secretion, with a similar pattern observed at the 6-month follow-up. Conversely, increased IL-10 secretion was observed in activated PBMCs from the patients without restenosis at baseline and follow-up, while no differences in IL-10 secretion were observed in the cells from patients with restenosis or angina (Figure 3).

## Discussion

Different publications have demonstrated that acute coronary syndrome can be characterized by unstable angina, non-ST-segment or ST-segment elevation myocardial infarction and chronic inflammation. It has also been suggested that an autoimmune mechanism could contribute to the atherosclerotic plaque progression and instability, where the oxidative modification of LDL played a pivotal role. Among others, these mechanisms include the increased expression of adhesion molecules on endothelial cells, monocyte activation, and pro-inflammatory cytokine secretion [15, 16]. We found that patients with recurrent ischemic events or restenosis had a predominant pro-inflammatory response and an increased percentage of total plaque area in the culprit vessel 6 months after PCI. This *ex vivo* response was similar to the *in vitro* response of PBMCs activated with nLDL. Positive peri-stent vascular remodeling has been associated with stent thrombosis due to late acquired stent malposition and this has been reported more frequent with drug eluting stent. This situation was identified in only two patients as the cause of recurrent ischemic event, but all our patients underwent to bare metal stent implantation. Two mechanisms are thought to contribute to the development of late acquired stent malposition: decreased plaque volume behind the stent due to clot lysis or plaque regression, and positive remodeling of the vessel wall [17].

In our study, the inflammatory response was measured by elevated C reactive protein levels, and this was associated with the recurrence of angina or restenosis at 6 months, similar to previous reports [1, 2, 12, 14–20]. We found a tendency toward higher serum concentrations of IL-8 in those patients with restenosis compared to those without restenosis, in agreement with previous reports that predominantly pro-inflammatory responses are associated with poor prognoses [21]. However, we also observed that patients with higher levels of IL-10 had reduced frequencies of restenosis. IL-10 has been shown to have a modulatory effect on the immune response (most likely on the Th2 phenotype); this response indirectly inhibits the secretion of cytokines from Th1 cells, which inhibits the expression of cellular adhesion molecules and stimulates the production of granulocyte-macrophage colony-stimulating factor, tissue factor and fibrinogen, as well as the proliferation of smooth muscle cells, cyclooxygenase 2 expression and cellular death [19–22]. By immunohistochemical analysis, Mallat et al. [23] demonstrated that low levels of IL-10 were associated with reduced nitric oxide production. Additionally, other reports have shown that low levels of IL-10 inhibit the production of tumor necrosis factor alpha and nitric oxide while simultaneously protecting the ischemic and reperfused myocardium by reducing the recruitment of neutrophils [24–28]. The levels of this anti-inflammatory cytokine (IL-10) could be protective by regulating the effect of pro-inflammatory cytokines, reducing the possibility that an unstable atherosclerotic plaque will form and resulting in an improved prognosis [25]. Some other cytokines, such as IL-4, IL-1 receptor agonist and TGF β1, have anti-inflammatory effects. However, until now, the role of TGF β1 has been unclear, as clinical studies of this factor reported inconsistent results, with some associating elevated serum levels of activated TGF β1 with increased severity of atherosclerotic coronary disease or recurrent acute ischemic events and others reporting the opposite. Other studies demonstrated that the administration of anti-TGF β1 antibodies reduced the neointimal lesion area and inhibited the differentiation of Th17 cells, which play a critical role in the formation of atherosclerotic plaques [27–29]. In our study, patients with recurrent ischemic events or restenosis tended to display higher levels of activated TGF β1, without an association with the number of vessels involved in the disease. It is likely that the secretion of activated TGF β1 leads to an increase in the production of extracellular matrix and to plaque progression and disruption [25, 29–33]. Moreover, we found elevated basal serum concentrations of activated TGF β1 and lower IL-10 levels in patients with recurrent angina events or restenosis of the target lesion at the 6-month follow-up. This finding suggests that a TH1 phenotype response before coronary intervention was associated with restenosis at follow up, characterized by an increase in the percentage of total plaque area and neointimal hyperplasia without changes in arterial remodeling. There was no significant difference in the type of immune response between patients with or without recurrent ischemic events at 6 months after PCI. During the acute phase of unstable angina, the immune response could have prognostic value for percutaneous coronary interventions.

We decided to compare the effects of native LDL from patients and healthy donors, taking into account the fact that patients had higher levels of lipid peroxidation products and different LDL molecule characteristics, with smaller molecules and higher concentrations of cholesterol esters than healthy donors. Our results demonstrated higher levels of PBMC proliferation in response to stimulation with nLDL from patients with coronary artery disease (LDLnP) compared to stimulation with nLDL from healthy donors (LDLnH). Moreover, we analyzed CD86 expression *ex vivo* and found that the PBMC from patients with restenosis had higher CD86 expression levels than those from patients without restenosis. When we analyzed the biological effect of cellular activation with the two types of nLDL, our results showed that LDLnP tended to result in increased expression levels of CD86. It has been demonstrated that the stimulation of antigen presenting cells with oxLDL increases the expression of CD86 and that its interaction with CD28 induces the activation and proliferation of T lymphocytes, as well as the secretion of cytokines such as IFN-γ [31, 32]. Furthermore, we found that the secretion of IL-1β was higher when PBMC were stimulated with LDLnP than with LDLnH. Previous reports have demonstrated that minimally modified LDL is capable of inducing the secretion of pro-inflammatory cytokines such as IL-1β and IL-6 [31a33]. However, we observed that LDLnH and LDLnP also induced IL10 secretion. Similar to our results, other studies have demonstrated that differentially modified forms of LDL induce RNA expression of IL10 and cytokine secretion, as a possible regulatory mechanism of cellular activation [34, 35].

## Conclusions

The patients with acute coronary syndrome and outward remodeling at the culprit lesion artery were found to have a predominantly pro-inflammatory response, with poorer responses to interventional coronary procedures and increased frequencies of restenosis. Restenosis was associated with an increase in the percentage of total plaque area and a recurrence of angina events after 6 months. In contrast, a predominantly anti-inflammatory response was associated with protection and an improved outcome. It appears that LDL has pro-atherogenic properties and induces CD86 expression as well as the secretion of cytokines like IL-1β, which participate in the atherosclerotic process.

## Abbreviations

oxLDL: Oxidized low-density lipoproteins
UA: Unstable angina
nLDL: Native low-density lipoprotein
PBMCs: Blood mononuclear cells
PCI: Intravascular ultrasound-guided percutaneous coronary intervention
IVUS: Intravascular ultrasound
EEM: External elastic membrane
CSA: Cross-sectional area
CRP: C-reactive protein
IL: Interleukin
TGF: Transforming growth factor.
